# Adenosine: an endogenous mediator in the pathogenesis of psoriasis*

**DOI:** 10.1590/abd1806-4841.20153689

**Published:** 2015

**Authors:** Moira Festugato

**Affiliations:** 1 Private medical office - Caxias do Sul (RS), Brazil

**Keywords:** Adenosine, Caffeine, Cyclic AMP, Methotrexate, Psoriasis, Th17 Cells

## Abstract

It is known that inflammatory and immune responses protect us from the invasion of micro-organisms and eliminate "wastes" from the injured sites, but they may also be responsible for significant tissue damage. Adenosine, as a purine nucleoside, which is produced in inflamed or injured sites, fulfills its role in limiting tissue damage. Although, it may have a pleiotropic effect, which signals it with a proinflammatory state in certain situations, it can be considered a potent anti-inflammatory mediator. The effects of adenosine, which acts through its receptors on T cell, on mast cell and macrophages, on endothelial cells, on neutrophils and dendritic cells, as they indicate TNF-alpha and cytokines, show that this mediator has a central role in the pathogenesis of psoriasis. The way it acts in psoriasis will be reviewed in this study.

## INTRODUCTION

Adenosine is an endogenous purine nucleoside that after being released from cells or being formed extracellularly it is diffused into surrounding cells where it binds to specific cell membrane structures called adenosine receptors.^1^ Studies have shown that adenosine attenuates inflammation in various disease models, acting through activation of its receptor.^2^ There are 4 types of receptors, all members of the family of receptors coupled to G protein. Genes for these receptors are designated A1, A2A, A2B and A3.^1^ Although adenosine is present in extracellular space at low concentrations, being metabolically in a "stressful" condition, it dramatically increases its extracellular levels.^1^ The role of adenosine as an extracellular signaling molecule was first established by Drury and Szent-Györgvi in 1929. Recent in vivo and in vitro studies confirmed the beneficial role of adenosine as an immunomodulator.^1^ First, adenosine is released in the vicinity of the immune cells in tissues subject to various forms of harmful stimuli, including ischemia and inflammation. Second, in most experimental systems, adenosine is immunosuppressive as a result of receptor occupation in various types of immune cells. Third, removing the signaling of endogenous adenosine exacerbates the immune activation and, consequently, aggravates the tissue dysfunction following acute damaging stimulus.^1,3,4^

## ADENOSINE MECHANISM OF ACTION AND REGULATION

**Figure 1:**
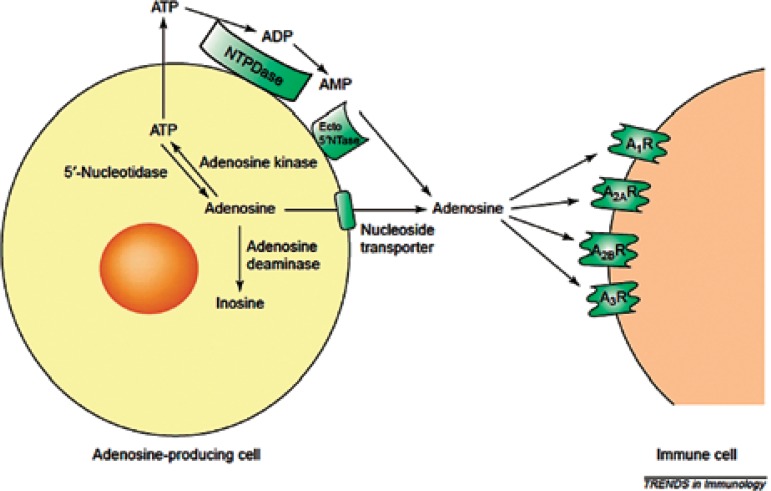
Mechanism of Action. Major pathway involved in the metabolism of adenosine. Adenosine is formed from its precursor ATP in both intra- and extracellular spaces. Intracellular adenosine is diverted into the extracellular space thourgh the nucleoside transporter. The enzyme adenosine kinase rephosphorylates adenosine to ATP as adenosine deaminase metabolizes adenosine to inosine. Extracellular adenosine formation is the result of an enzymatic cascade consisting of NTPDase and ecto-5’-nucleotidase (Ecto5’NTase). Extracellular adenosine binds to receptors A1, A2A, A2B, A3, which are expressed on the surface of immune cells.^1^

Source: Haskó G et al, 2004.^1^

The way adenosine regulates the immune system is through its bioavailability at the receptor site.^1^ A good example is hypoxia or tissue ischemia situations, which increases intracellular adenosine through purinergic metabolic pathway. Under these conditions occurs the dephosphorylation of ATP (adenosine triphosphate) by the enzyme 5-nucleotidase, while in parallel occurs the suppression of adenosine kinase enzyme activity, preventing the rephosphorylation of the adenosine.^1^ Once reaching high concentrations inside the cell, adenosine is then diverted into the extracellular space by a nucleoside transporter.^1^ Another pathway, and probably the dominant one, which contributes to high levels of extracellular adenosine, is comprised by the release of adenine nucleotides precursors (ATP = adenosine triphosphate, ADP = adenosine diphosphate AMP = adenosine monophosphate) from the cell to be metabolized to adenosine in the extracellular space by enzymes such as CD39 (dephosphorylase nucleoside triphosphate [DNTP]) and CD73 (5'ectonucleotidase).^1,3^ Adenosine bioavailability is limited by its catabolism to inosine by adenosine deaminase enzyme (ADA), which is further degraded to uric acid, the final stable compound (Figure 1).^1^

## IMPLICATION OF ADENOSINE DEAMINASE ENZYME (ADA) IN PSORIASIS 

ADA is widely found in human tissue, having a greater activity in the lymphoid tissue. It is associated mainly with T lymphocytes.^5^ Studies show increased levels of ADA in diseases characterized by proliferation or activation of T cells. In the study of Bukulmez, the authors found normal values of ADA in serum of patients with psoriasis before treatment, but significantly higher when compared with the control group.^5^ ADA activity decreased after treatment with PUVA and cyclosporine, showing that ADA activity is associated with activation of T cells. There was no correlation between ADA levels and disease activity compared with PASI score. This may result from a lack of objectivity of PASI scoring system for estimating the disease activity.^5^ The high activity of this enzyme in patients with psoriasis before treatment and its reduction after treatment shows that ADA activity is related to disease activity. More studies are needed to determine its sensitivity in the follow up of the disease and in the prevention of relapses before clinical findings.^5^

## IMPLICATION OF ADENOSINE IN MAST CELLS AND MACROPHAGES IN PSORIASIS PATHOGENESIS

In psoriasis, other trigger factors produce lesions that are clinically and histologically identical and have a common final pathway, which is believed to involve activation of macrophages.^6^ Macrophages, in turn, contain TNF-alpha, what explains how a diverse group of stimuli could initiate the cascade of cytokines necessary for induction of psoriasis phenotype. In psoriasis lesion there is a close association between mast cells that release histamine and macrophages containing TNF-alpha.^6^ Mast cells, with respect to adenosine, is an exception to the rule.^1^ By the occupation of A2B and A3 receptors, they cause degranulation with the release of histamine. The number of mast cells is increased in psoriatic lesions, mainly in the subepidermal connective tissue.^7^ There are evidence that histamine release via adenosine receptors promote a negative feedback, signaling TNF-alpha production by macrophages, and then inflammation.^1^ Furthermore, mast cells alone can activate other mediators that can affect the immune system and promote inflammation.^7^ Psoriasis patients present an increase in the expression of a factor called "stem cell factor (SCF) kit ligand", responsible for the migration, activation, and maturation of mast cells.^7^

## ADENOSINE IN T CELL REGULATION FUNCTION (Tregs)

(Tregs) Recent studies have reported that adenosine is a significant mediator of T cell regulatory function (Treg) by the action in A2A receptor.^2^ Activation of the receptor in various immune and inflammatory cells attenuates the inflammation. This anti-inflammatory effect is associated with increased intracellular cAMP, which inhibits cytokines, including T cells and antigen presenting cells (APCs). Adenosine regulates T cell function indirectly by reducing the secretion of cytokines, including IL-12, TNF-alpha and other cytokines, which have their production inhibited via A2A receptor; IL-2, IL-4, IFN-gamma and TNF-alpha (90%).^2^

The first evidence that adenosine plays a role in regulating the biological function of T cells (Tregs) came from a study in mice that prevented the induction of colitis by effector Th cells (Teffs) by acting on A2A receptors. In the effector Th cells that didn't have receptors, the study failed to control colitis, suggesting that adenosine contributed to Treg for the anti-inflammatory response. Another additional evidence came from experiments in which the presence of Foxp3 is positively associated with (ecto-NTPDase-1 [CD39]). The ability of Tregs to convert ATP to adenosine proves the direct evidence of the functional capacity of this enzyme and supports the notion that adenosine mediates T cells regulatory function.^2^

In these studies, it was also observed that Treg uses different mediators, in addition to adenosine, with different effects on Teff function. Treg, through direct contact with the target cell, transfers cAMP to Teff. This is shared by the TGF-B1 molecule associated with the membrane and causes a more suppressive effect.^2^ Regardless of what was said, adenosine can also cause diverse response. When TGF-B1 acts alone, the response is anti-inflammatory, but when in combination with IL-4 or IL-6, it stimulates Th9 and Th17. Inhibition of IL-12 bound to IL-6 induction in dendritic cells converts the anti-inflammatory potential of TGF-B1 in proinflammatory that induces Th17.^2^

Th17 is a subclass of T cell analyzed recently, distinct from Th1 and Th2 cells, which have been implicated in the pathogenesis of psoriasis and other inflammatory diseases.^3^ IL-23 is a regulatory cytokine in these diseases and stimulates the proliferation and maturation of Th17. In the psoriasis lesions, the production of IL23 by dendritic cells and keratinocytes is increased, stimulating Th17 cells within the dermis to produce "Th17 cytokines", which includes IL-17A, IL-17F, TNF-alpha, IL-21, and IL-22. IL-22 is a potent stimulator of keratinocyte proliferation and has gained prominence in the pathogenesis of psoriasis as a key cytokine effector of Th17.^4^

## IMPLICATION OF ADENOSINE IN THE MIGRATION T LYMPHOCYTE FROM PERIPHERAL VESSELS TO SKIN IN PSORIASIS PATHOGENESIS

In psoriasis the migration of T lymphocytes from peripheral vessels to skin is dependent on adhesion molecules.^8^ Endothelial cells express adhesion molecules, which are responsible for the recruitment of leukocytes at inflamed sites.^1^ Endothelial cells also synthesize and release mediators such as platelet activating factor, IL-8 and IL-6, which has a direct role in the inflammatory process for conducting the movement of leukocytes between tissue compartments.^1^ Expression of A2A and A2B adenosine receptors on endothelial cells of various types is well documented.^1^ Cells that release most extracellular adenosine are endothelial cells and neutrophils. Endothelial cells are a source of adenosine by its phosphorylation capacity of adenine to adenosine with activation of the classical pathway Gs-cAMP. The interaction of neutrophils with endothelial cells involves the action of ecto-5›nucleotidase, promoting an endothelial barrier action through A2B receptor.^1^ Adenosine may inhibit the release of IL-6, IL-8, and adhesion molecules (E-selectin and VCAM 1) by action of A2A receptors.^1^ Among the adhesion molecules, E-selectin is the most important, being the one that controls migration. Others such as ICAM 1, ICAM 2, VICAM 1 are the key to endothelial adhesion and also to keratinocyte-leukocyte interaction.^9^ Adenosine fulfills its anti-inflammatory role, preventing that the inflammatory process is perpetuated. Nevertheless, if it is perpetuated, adenosine can stimulate the release of IL-8 by acting in A2B receptors due to activation of phospholipase C through "Gq protein" pathway, and not through the classical pathway.^1^ IL-8 stimulates the chemotaxis of neutrophils and T cells and the proliferation of keratinocytes.^6^ Neutrophils promote the disruption of desmosomes of keratinocytes and formation of Munro's microabscesses, while maintaining the differentiation of T cells to Th1.^9^

## INTERACTION OF ADENOSINE RECEPTOR WITH ANTIGEN PRESENTING CELLS (APCs)

Macrophages and dendritic cells (DCs) are specialized phagocytes, responsible for the "cleansing" of apoptotic cells and harmful molecules, as well as for the defense against infections. These antigen presenting cells (APCs) are widely dispersed in the body, including microorganisms gateways.^1^ They also participate in the initial capturing and processing of antigens and in the activation of a lymphocyte effector mechanism. These activated lymphocytes cooperate with macrophages to increase pathogens destruction.^1^ Dermal monocytes, macrophages and dendrocytes are a collection of heterogeneous cells that constitute the phagocytic system of the skin, and two cell lines can be distinguished: monocyte-macrophage and dendritic cell. The latter cell line includes, among others, Langerhans cells and dermal dendrocytes.^10^

In the psoriasis lesions, dermal dendritic cells (DDC) are increased in number, while in the epidermis, dendritic cells (especially Langerhans cells) are decreased.^11^

In psoriasis, dendritic cells (DC) and Langerhans cells (LC) promote T cell proliferation and cytokine release.^12^ LC is diminished in plaque psoriasis (both in density and in its intraepidermal distribution). These cells present ATPase activity and have an anti-inflammatory role for degrading pro-inflammatory ATP and their decrease in plaque psoriasis can prolong the inflammatory reaction.^12^

Probably the most important recent development on the role of APCs is the discovery of pattern recognition receptors (PRRs), which allows the recognition of conserved repetitive microbial elements, generally lipopolysaccharides (LPS), CpG DNA, and viral RNA. These include, among others, Toll-like receptors (TLRs).^1^ Signals initiated by the occupied adenosine receptor may interfere with the intracellular pathway activated by PRRs. A2A receptors decrease the induced release via TLR4 of other pro-inflammatory mediators, including TNF-alpha, and increase the production of IL-10, an anti-inflammatory cytokine. A2A and A3 receptors are involved in the suppression of pro-inflammatory mediators following TLR stimulation. The adenosine receptor bound to monocytes and macrophages strongly suppresses the production of IL-12 induced by LPS through TLR4. IL-12 is the cytokine that guides to a strong inflammatory response and adenosine suppresses its production, this is the main mechanism by which adenosine receptor occupancy suppresses inflammation of the injured tissue.^1^

Consistent with this retroregulation of IL-12 production by mature DCs, in the presence of adenosine, DCs have an decreased ability to promote differentiation of T cells to Th1. Reduction in this differentiation constitutes another mechanism by which adenosine suppresses inflammation, because Th1 cells are strong inducers of macrophage mediated inflammatory response.^1^

## ADENOSINE AND METHOTREXATE

Methotrexate is the most widely used immunosuppressant in psoriasis.^1^

Methotrexate is a folic acid analogue capable of inhibiting, competitively and irreversibly, the enzyme dihydrofolate reductase. Therefore, the conversion of dihydrofolate to tetrahydrofolate -a cofactor required for transferring carbon atoms, essential for the synthesis of DNA and RNA -doesn't occur. It also acts by inhibiting, in a partially reversible way, the thymidylate synthase enzyme, which is involved in cell proliferation.^13^

In 1951, the first effects of the drug in psoriasis were observed, and it was approved by the FDA as standard treatment in 1971. Initially, it was believed that its effect elapsed only from the activity on the proliferation of keratinocytes (antiproliferative property), but more recently new fronts of action were discovered: the action of methotrexate on lymphocytes and its effect on adenosine, a potent anti-inflammatory mediator.^13^

The drug acts on the metabolism of adenosine, causing its accumulation. The excess adenosine, in turn, binds to A2A receptor in endothelial cells, inhibiting apoptosis, chemotaxis of neutrophils and release of TNF-alpha, IFN-gamma, IL-12, and IL-6. This results in its anti-inflammatory activity. After an hour of ingestion, distribution and cellular uptake is complete.^13^

Another action of methotrexate is to induce an increase in adenosine release from damaged cells by selective inhibition of AICAR (5-aminoimidazole-4-carboxamidoribonucleotide) transformylase, an enzyme that catalyzes an intermediate reaction in purine biosynthesis, and the released adenosine inhibits inflammation.^1^

**Figure 2:**
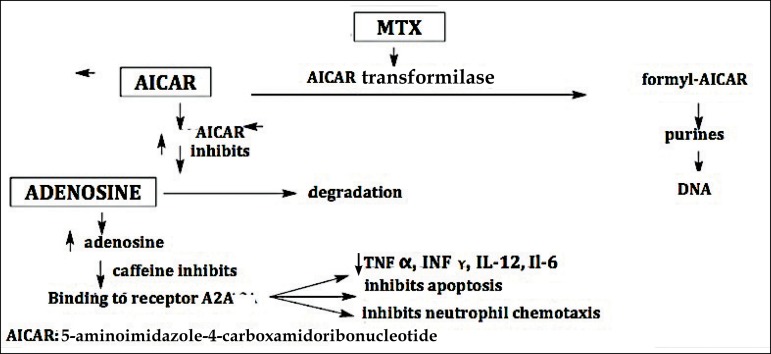
Schematic illustration of methotrexate action

Source: Bressan, et al 2010.^14^

Methotrexate also inhibits the adenosine deaminase enzyme (ADA).^14^ Data suggest that inhibition of both, AICAR transformylase and ADA, is connected to efficacy of low doses of methotrexate (Figure 2).^14^

Co-administration of methotrexate and caffeine reverses the anti-inflammatory effects of methotrexate in patients with arthritis-type inflammation related with antagonist effect of caffeine on adenosine receptors interfering with the effects of methotrexate.^3,1,5^

Decaffeinated coffee also interferes with methotrexate anti-inflammatory action.^1^ In Brazil, one of the most popular and widely consumed brands of decaffeinated coffee has a maximum content of 0.3% caffeine. According to Agência de Vigilância Sanitária (ANVISA), the maximum of caffeine in a decaffeinated product in g/100 mg should be 0.1%.^16^ It is recommended to avoid caffeine, which could increase the therapeutic effect of methotrexate.^1^

Caffeine is a thermogenic food that should be avoided in patients with psoriasis.^17^ In this pilot study, 43 patients were evaluated over 2 years, and 88.37% reached positive results with the suspension of this kind of food and with the changing of eating habits, such as: reduction of scales and erythema, milder outbreaks, more delay to the onset of lesions during the year and improved quality of life.^17^

## CONCLUSION

The objective of this review is to bring to knowledge this adenosine mediator, which was considered as unimportant, after the discovery of cytokines in the pathogenesis of psoriasis. We observed that adenosine is involved in immune mechanisms of psoriasis and in gene expression. Although, we do not know the primary defect that triggers it, the fact that adenosine acts signaling macrophages and mast cells, which according to this study, have an initial role in the development of lesions and, on the other hand, we found that adenosine indirectly regulates the function of T cells by reducing the secretion of cytokines so that does not perpetuate inflammation, places this mediator as co-participant in the process, hence its pleiotropic effect. Regarding psoriasis, there is the need to promote the "mitigation" of this mediator and contain the "transgressor". It's important to perform a comprehensive history, elucidating aspects of lifestyle of the patient, his/her habits, alimentation and addictions (alcohol, tobacco, energy drinks, etc.). Also, it's significant to treat patient's infections, as recurrent streptococcus infections, before beginning any therapy. This is, don't increase any further the notorious inflammatory contingent that already exists in these patients, allowing that the host's own defenses can act in his/her favor, and not against him/her. We must consider comorbidities related to psoriasis, in which adenosine action is also present. Regarding comorbidities, it is clear that a disease increases the vulnerability of the other, then, adopting simple measures, such as guiding the patient to a healthier lifestyle, working in multidisciplinary team (physicians, psychologists and nutritionists), these measures could lead to more economical therapies that treat the underlying cause of the disease, while lowering adverse events of drugs employed.
